# New Insights into YAP/TAZ-TEAD-Mediated Gene Regulation and Biological Processes in Cancer

**DOI:** 10.3390/cancers15235497

**Published:** 2023-11-21

**Authors:** Yang Zhao, Marisela Sheldon, Yutong Sun, Li Ma

**Affiliations:** 1 Department of Experimental Radiation Oncology, The University of Texas MD Anderson Cancer Center, Houston, TX 77030, USA; yzhao18@mdanderson.org (Y.Z.); mesheldon@mdanderson.org (M.S.); 2 Department of Molecular and Cellular Oncology, The University of Texas MD Anderson Cancer Center, Houston, TX 77030, USA; ysun2@mdanderson.org; 3 The University of Texas MD Anderson Cancer Center UTHealth Houston Graduate School of Biomedical Sciences, Houston, TX 77030, USA

**Keywords:** YAP, TAZ, TEAD, gene regulation, metastasis, cancer therapy

## Abstract

**Simple Summary:**

The Hippo pathway is crucial for regulating cell growth, organ size, and tissue regeneration. When this pathway goes awry, it can lead to uncontrolled cell growth, tumor spread, and resistance to cancer treatments. This review covers recent progress in understanding how the Hippo pathway is involved in cancer and discusses potential therapies targeting this pathway. In addition, we address ongoing debates and provide perspectives on debated topics in this field.

**Abstract:**

The Hippo pathway is conserved across species. Key mammalian Hippo pathway kinases, including MST1/2 and LATS1/2, inhibit cellular growth by inactivating the TEAD coactivators, YAP, and TAZ. Extensive research has illuminated the roles of Hippo signaling in cancer, development, and regeneration. Notably, dysregulation of Hippo pathway components not only contributes to tumor growth and metastasis, but also renders tumors resistant to therapies. This review delves into recent research on YAP/TAZ-TEAD-mediated gene regulation and biological processes in cancer. We focus on several key areas: newly identified molecular patterns of YAP/TAZ activation, emerging mechanisms that contribute to metastasis and cancer therapy resistance, unexpected roles in tumor suppression, and advances in therapeutic strategies targeting this pathway. Moreover, we provide an updated view of YAP/TAZ’s biological functions, discuss ongoing controversies, and offer perspectives on specific debated topics in this rapidly evolving field.

## 1. Introduction

Originally discovered in *Drosophila melanogaster*, genetic alterations in the Hippo pathway have been found to cause overgrowth [1,2,3,4,5]. Subsequent studies revealed that this pathway is conserved across species, spanning from flies to humans. The core kinases of the mammalian Hippo pathway, including mammalian Ste20-like 1/2 (MST1/2) and large tumor suppressors (LATS1/2), function to restrain cellular growth by inactivating two transcriptional coactivators, Yes-associated protein (YAP) and WW domain-containing transcription regulator protein 1 (TAZ) [6,7,8]. A growing body of research has advanced our understanding of the roles of Hippo signaling in various human cancers, as well as its functions in development and tissue regeneration. Moreover, extensive exploration in recent years has provided new insights into the molecular complexities, regulatory mechanisms, and potential therapeutic targets of this pathway [7,8,9,10].

The Hippo pathway consists of two main modules: the upstream MST1/2-LAST1/2 kinase complex, responsible for integrating intrinsic and extrinsic signals, and the downstream YAP/TAZ-TEA domain transcription factor (TEAD) complex, serving as the effector for driving the expression of target genes [11]. In response to a variety of stimuli, MST1/2, in conjunction with its binding partner Salvador homolog 1 (SAV1), phosphorylate and activate LATS1/2 at specific threonine residues (Thr1079 on LATS1 and Thr1041 on LATS2 in humans). This kinase activation extends to their co-activators, MOB kinase activator 1A/B (MOB1A/B), at Thr35 in humans. Subsequently, LATS1/2 phosphorylate YAP/TAZ at evolutionarily conserved serine residues located at the N-terminus of these proteins (Ser127 in human YAP and Ser89 in human TAZ). Upon phosphorylation, YAP/TAZ are recognized by 14-3-3 protein, leading to their sequestration and inactivation in the cytoplasm and/or degradation by the proteasome [7,12,13,14]. In the absence of the active Hippo kinase cascade (Hippo off), YAP/TAZ translocate to the cell nucleus. Within the nucleus, YAP/TAZ, which contain transactivation domains but lack DNA-binding domains, associate with TEAD1-4 (containing DNA-binding domains but lacking transactivation domains) to activate the expression of proproliferative and prosurvival genes [15,16,17].

Over the past decades, emerging evidence has underscored the roles of YAP/TAZ in promoting human cancers, including liver, lung, breast, brain, pancreas, and skin malignancies [7,13]. Analyses of The Cancer Genome Atlas (TCGA) data have indicated that the Hippo pathway is one of the most frequently altered signaling pathways in cancer [18]. Elevated YAP/TAZ activity has been implicated in regulating multiple hallmarks of cancer, including the maintenance of proliferative signaling, initiation of invasion and metastasis, deregulation of cellular metabolism, and evasion of immune surveillance [19,20,21,22]. Tumors harboring dysregulated components of the Hippo pathway are not only resistant to intrinsic cell death mechanisms but also exhibit increased tolerance to chemotherapy, radiotherapy, and targeted therapy.

Recent studies continue to reveal new facets of YAP/TAZ’s role in driving malignancies and open new avenues for therapeutic interventions. For instance, cancers can manifest as YAP-activated (YAP^on^) or YAP-deactivated (YAP^off^) states [23]. This finding adds layers of complexity to our understanding of YAP/TAZ, indicating its context-dependent regulation by both intrinsic genomic factors and extrinsic stressors. These nuances pose new challenges and questions for future research. Meanwhile, studies of fundamental molecular mechanisms have paved the way for clinical translation. Emerging pharmacological inhibitors targeting the YAP/TAZ-TEAD pathway have been developed to promote efficacy and counteract drug resistance [7,8,9].

In this review, we discuss recent studies concerning YAP/TAZ-TEAD-mediated gene regulation and biological processes in the context of cancer. The knowledge gained from these studies provides new insights into the complexity of cancer and could lead to the development of more effective therapeutic strategies.

## 2. Advances in Upstream Regulators of Hippo Signaling

**Figure 1:**
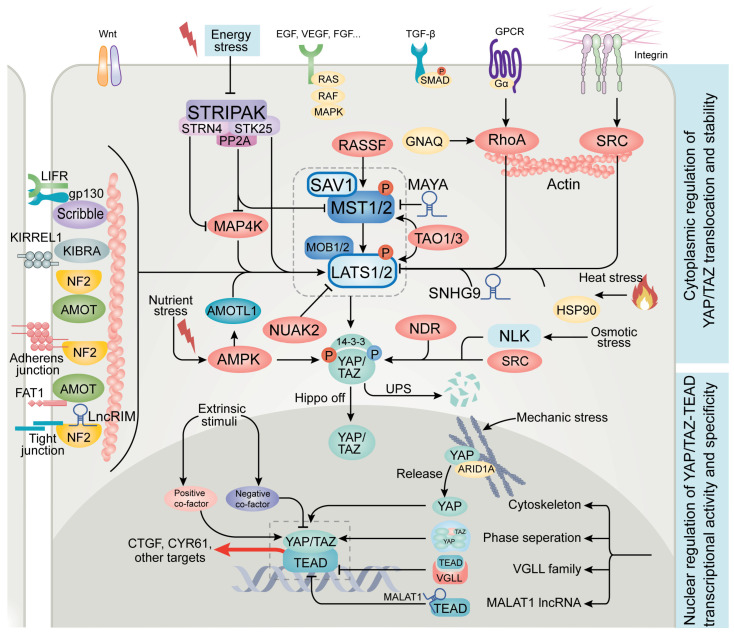
Regulators of YAP/TAZ in the cytoplasm and the nucleus. In the cytoplasm, Hippo pathway components act as sensors and integrators of signaling inputs. In the nucleus, YAP/TAZ-TEAD and other cofactors act as effectors to control gene transcription. Various inputs, such as osmotic stress, heat shock, nutrient stress, mechanic stress, cell–cell contact, and cell polarity, are transmitted to YAP/TAZ-TEAD in Hippo-dependent and independent manners. Nutrient stress activates AMPK, which phosphorylates and inactivates YAP/TAZ; AMPK also phosphorylates and stabilizes AMOTL1, which in turn aids LATS-mediated YAP/TAZ dephosphorylation and inactivation. Heat shock promotes LATS dephosphorylation through HSP90 and PP5, while concurrently fostering LATS ubiquitination and degradation to activate YAP/TAZ. Osmotic stress induces transient nuclear translocation of YAP, a process facilitated by NLK, which phosphorylates YAP at Ser128, disrupting its interaction with 14-3-3. Cell adhesion molecules and their intracellular adaptors, such as FAT1, KIRREL1, NF2, AMOT, KIBRA, and Scribble, anchor MST and LATS to the plasma membrane to sustain their ability to inactivate YAP/TAZ. Stimulation of GPCRs can result in either activation or inhibition of YAP/TAZ, depending on the coupled G protein. Mechanic force, ECM stiffness, and shear stress exert on integrins and cause LATS1/2 inactivation through SRC, while SRC can also phosphorylate and activate YAP/TAZ, promoting their nuclear translocation. Cytoskeleton remodeling proteins, such as RhoA, are instrumental in LATS inactivation and cooperate with other signaling molecules including GNAQ, GPCR, and others. The STRIPAK complex is important in initiating MST-LATS activation through its multiple components, including STRN4, STK25, and PP2A. MAP4K is regulated by the STRIPAK complex and activates LATS1/2 in parallel to MST1/2 activation. TAO1/3 kinases promote LATS activation in both MST-dependent and -independent manners. NUAK2 acts as a YAP/TAZ activity enhancer by antagonizing LATS-mediated YAP/TAZ phosphorylation. Certain cytoplasmic lncRNAs, such as MAYA, SNHG9, and LncRIM, regulate Hippo pathway components by acting as scaffolds. The nuclear regulations of YAP/TAZ-TEAD include: (1) nuclear mechanical stress enhances the interaction of F-actin with the ARID1A-SWI/SNF complex, leading to the liberation and activation of YAP/TAZ; (2) YAP, TAZ, and their fusion proteins can undergo phase separation, facilitating their transcriptional activity by chromatin remodeling; (3) VGLL family members sequester TEAD to inhibit YAP/TAZ-TEAD binding, restricting transcriptional activity; and (4) the nuclear lncRNA MALAT1 binds and sequesters TEAD to block its interaction with YAP and target gene promoters. Other signaling pathways, such as RAS-RAF-MAPK, TGF-β–SMAD, GPCR, integrin, and Wnt pathways, interact with the Hippo pathway to exert positive or negative effects on the activity of YAP/TAZ.

Accumulating evidence has revealed that the activity of Hippo pathway components depends on their proximity to the plasma membrane [24]. The subcellular localization of these molecules is coordinated through a range of mechanisms involving membrane receptors, adaptor proteins, and RNAs [8] (Figure 1). A pivotal event in Hippo signaling is the activation of MST and LATS kinases. Among the proteins orchestrating this process, scaffold proteins like WW domain-containing protein 1 (WWC1, also known as kidney and brain protein (KIBRA)) and neurofibromin-2 (NF2, also known as merlin) facilitate the recruitment of MST1/2 to the plasma membrane. Subsequently, LATS1/2 associates with the MST1/2-MOB1A/B complex, leading to LATS1/2 phosphorylation and activation by MST1/2 [25,26]. Additional proteins, including angiomotin (AMOT) [27,28], Kin of IRRE-like protein 1 (KIRREL1) [29,30,31], FAT1 [32], E-cadherin [33], leukemia inhibitory factor receptor (LIFR) [34], and Scribble [35], play similar roles by anchoring MST and LATS to the plasma membrane to sustain their activities. Notably, alterations of these proteins, such as NF2, E-cadherin, AMOT, and FAT1, are often observed in cancer and are associated with elevated YAP/TAZ activity [32,36,37].

Ras association domain-containing protein (RASSF) is recognized as a family of core Hippo pathway scaffolds that interact with MST1/2 through a mechanism similar to SAV1. The RASSF family comprises proteins encoded by genes *RASSF1-10*, with RASSF1-6 containing a SARAH domain that enables binding to MST1/2. Among the family members, RASSF1 and RASSF5 have been studied most [38]. RASSF1A, in particular, plays a crucial role in the activation of MST kinases by directly binding to them, thus promoting MST1/2 kinase activity, enhancing the MST-LATS interaction, and stabilizing the active, phosphorylated forms of MST kinases by preventing dephosphorylation [39]. Moreover, RASSF1A facilitates the formation of a YAP-p73 complex in the nucleus, leading to enhanced transcription of the pro-apoptotic gene *PUMA* [40]. In line with its tumor-suppressive role, RASSF1A is frequently inactivated through hypermethylation of promoter regions in various cancer types, such as liver, breast, and lung cancers. Notably, the expression levels of RASSF1A are associated with increased cancer risk and poor clinical outcomes [41].

Recent studies have also demonstrated the involvement of long noncoding RNAs (lncRNAs) in regulating Hippo signaling. For instance, the lncRNA MST1/2-antagonizing for YAP activation (MAYA) facilitates MST1 methylation at Lys59, which inhibits MST1’s kinase activity, leading to LATS1 inactivation. This regulatory effect is mediated by the interaction between MAYA, the adaptor protein LLGL scribble cell polarity complex component 2 (LLGL2), and the methyltransferase NOL1/NOP2/Sun domain family member 6 (NSUN6) [42]. Small nucleolar RNA host gene 9 (SNHG9), another lncRNA, binds to LATS1’s C-terminal domain, instigating LATS1 phase separation and impeding YAP phosphorylation by LATS1 [43]. Moreover, LncRNA related to iron metabolism (LncRIM), also known as ZBED5-AS1 and Loc729013, directly interacts with NF2, thus preventing NF2 from binding to LATS1, which leads to YAP activation and the modulation of intracellular iron metabolism [44]. The pivotal role of lncRNAs in mediating protein–protein interactions [45] suggests the likelihood of unveiling additional lncRNAs governing the activation of the MST-LATS kinase complex in future research.

In addition to MST1/2, other kinases and certain phosphatases have been found to modulate LATS1/2 activity. Thousand and one amino acid protein 1/3 (TAO1/3), for instance, directly phosphorylates LATS1/2 independently of MST1/2 [46]. Moreover, TAO1 can also phosphorylate and activate MST2 [47,48], suggesting that TAO proteins influence LATS activation in MST-dependent and -independent manners. The mitogen-activated protein kinase kinase kinase kinase (MAP4K) family, functions in parallel—and sometimes redundantly—with MST1/2 to phosphorylate and activate LATS1/2 [49]. This is modulated by striatin-4 (STRN4), a striatin-interacting phosphatase and kinase (STRIPAK) complex component, which binds and inhibits MAP4K, thus promoting YAP activation [50,51]. Intriguingly, the STRIPAK complex has been identified as a regulator of Hippo signaling through genomic and proteomic approaches [52,53]. Serine/threonine-protein phosphatase 2A catalytic subunit C (PP2AC), the phosphatase catalytic subunit of the STRIPAK complex, associates with MST1/2 and MAP4K4, inactivating them through dephosphorylation [54]. In addition, another STRIPAK component, striatin-3 (STRN3), recruits MST1/2 to PP2A, promoting MST1/2 dephosphorylation. Notably, elevated levels of STRN3 correlate with increased YAP activity and poor clinical outcomes in gastric cancer [55]. Serine/threonine-protein kinase 25 (STK25), a kinase associated with the STRIPAK complex, has dual roles: phosphorylating and activating LATS1/2 [56] and phosphorylating SAV1 to lessen its inhibitory effect on the STRIPAK complex [57,58]. Recently, Seo et al. [59] reported that energy stress disrupts the MAP4K2-STRIPAK association, leading to the activation of MAP4K2, which in turn phosphorylates the autophagy-related protein 8 (ATG8) family member LC3, thereby facilitating autophagic flux. Taken together, these studies underscore the significance of the TAO kinase family and the STRIPAK phosphatase complex in regulating Hippo signaling, particularly under stress conditions.

Besides LATS1/2, YAP/TAZ activity is influenced by other factors that function in parallel to the classic Hippo kinase cascade. Notably, AMP-activated protein kinase (AMPK) directly phosphorylates YAP at multiple sites, particularly Ser61 and Ser94. This disrupts the interaction between YAP and TEAD, thereby inhibiting the transcription of their target genes [60,61]. AMPK also phosphorylates and stabilizes angiomotin-like protein 1 (AMOTL1), which in turn aids LATS-mediated YAP phosphorylation and inactivation [60,62]. Interestingly, recent findings identified NUAK family SNF1-like kinase 2 (NUAK2), an AMPK family member, as a YAP/TAZ activity enhancer, which functions by antagonizing LATS-mediated YAP/TAZ phosphorylation [63]. Furthermore, *NUAK2* itself is a target gene of the YAP/TAZ/AP-1 complex, and its expression is crucial for robust YAP/TAZ signaling, forming a positive feedback loop [63,64]. Additional AMPK family members, such as serine/threonine-protein kinases (SIKs) and Par-1/MARKs, have been identified as either positive or negative regulators of the Hippo pathway [65,66,67,68,69], while the in vivo functional relevance warrants further validation. In addition to LATS and AMPK, mammalian NDR kinases (NDR1/2) can also phosphorylate YAP at S127, as evidenced by studies involving NDR1/2-deficient mice [70]. Remarkably, NDR kinases are the closest homologs to LATS kinases [71], underscoring the importance of S127 phosphorylation in the regulation of YAP activity. Collectively, these studies suggest that cells have evolved redundant mechanisms to regulate YAP.

Cytoskeletal remodeling proteins, such as the SRC family kinases (SFKs), mainly SRC and YES, and also Rho GTPases, notably RhoA, hold significant sway in the modulation of YAP/TAZ activity through both Hippo-dependent and -independent mechanisms. RhoA is a member of the Rho family of GTPases and primarily functions to regulate the organization and dynamics of the actin cytoskeleton. Dupont et al. [72] were the first to report that YAP/TAZ operate downstream of RhoA, responding to mechanical cues derived from the extracellular matrix (ECM) rigidity and changes in cell shape. Moreover, Plouffe et al. [46] engineered a series of knockout cell lines for many components of the Hippo pathway, revealing RhoA and NF2 as key regulators of YAP/TAZ activity. Additional evidence has demonstrated that RhoA modulates LATS1/2 activity independently of MST1/2 by coordinating actin polymerization and stress fiber formation in response to mechanical stimuli [46,72,73,74]. Furthermore, RhoA cooperates with other signaling molecules, including G protein-coupled receptor (GPCR) [73], Wnt [75], protein kinase A (PKA) [76], Ras-related protein Rap-2 (RAP2) [77], guanine nucleotide-binding protein alpha-q (GNAQ) [78], and p53 [79], under specific stress conditions [61,80] to regulate YAP/TAZ activity. For example, Yu et al. [73] demonstrated that GPCR signaling can either activate or inhibit LATS1/2 in a RhoA-dependent manner. Serum-borne mitogens, such as lysophosphatidic acid (LPA) and sphingosine 1-phosphate (S1P), activate Gα_12/13_ or Gα_q/11_, which in turn triggers RhoA activation and subsequent actin dynamics, leading to LATS1/2 inactivation. On the other hand, stimulation of Gs-coupled GPCRs by glucagon or epinephrine inhibits Rho GTPase via activation of PKA, attenuating actin dynamics and consequently suppressing YAP/TAZ activity [73,76]. In addition, *GNAQ* and *GNA11* encode the proteins Gαq and Gα_11_, respectively. These genes are known to function as driver oncogenes in uveal melanoma, with over 80% of cases carrying mutations in *GNAQ* and *GNA11*. Gain-of-function mutations in GNAQ stimulate YAP through a signaling pathway regulated by RhoA and Rac1. This not only leads to LATS inactivation but also causes actin remodeling by RhoA, disrupting the binding of YAP to AMOT. Consequently, YAP is released and translocates to the nucleus [78]. Interestingly, RhoA can act downstream of specific metabolic pathways, such as the mevalonate pathway. Mevalonate serves as a precursor for geranylgeranyl pyrophosphate (GGPP), which promotes membrane localization and activity of Rho GTPases, leading to nuclear localization and activation of YAP/TAZ [80]. These findings underscore the intricate role of Rho GTPases in the regulation of YAP/TAZ, while the specific mechanisms by which RhoA integrates diverse upstream signals, as well as its interplay with cytoskeletal remodeling, await further elucidation.

SRC mainly functions as an effector of the integrin pathway, integral to the response to ECM stiffness and mechanical cues. SRC family members govern YAP/TAZ in both Hippo-dependent and -independent manners. SRC and YES directly phosphorylate YAP1 (primarily at Y341, Y357, and Y394) independently of the Hippo pathway, activating YAP/TAZ without affecting LATS1/2 activity or YAP localization [81,82,83]. This phosphorylation event manifests downstream of multiple pathways, such as IL6-gp130 and Wnt–β-catenin pathways, engendering the activation of YAP/TAZ without concomitant inactivation of LATS1/2 or perturbation of YAP distribution [82,84]. SRC can also be modulated by alternative upstream regulators, such as focal adhesion kinase (FAK), which leads to the dephosphorylation and inactivation of LATS1/2 [85,86]. Furthermore, Rho GTPases and SRC kinases engage in extensive crosstalk concerning cytoskeletal regulation [87,88]. Their indispensable roles in the regulation of YAP/TAZ underscore the intricate convergence of diverse upstream signals. This concerted action operates to dynamically modulate downstream effectors in a temporally responsive manner.

Cells inhabit a dynamic milieu, continuously exposed to a variety of physical, chemical, and biological factors. The Hippo pathway embodies a multifaceted regulatory framework that reacts to a wide spectrum of stimuli, including cell–cell contact [89,90], cell polarity [91], temperature fluctuations [92], mechanic forces [72,77], osmotic stresses [93,94], oxygen levels [95,96], nutrient availability [80,97,98], ions [44], and the presence of other cells or bioactive molecules [99,100,101]. It has been well recognized that both cell density and serum deprivation impact YAP activity in cell culture. Cell density-mediated regulation mainly operates through cell–cell contacts, inclusive of both adherent and tight junctions [90]. Serum deprivation, on the other hand, exerts its effects through serum-derived factors such as lysophosphatidic acid (LPA) and sphingosine-1-phosphate (S1P) [73]. Nutrient stress triggers AMPK activation, which restrains YAP/TAZ through both Hippo-dependent and -independent pathways, thereby highlighting a cell survival strategy that prioritizes energy allocation through rapid kinase cascade activation [60,61,62,102]. Recent work has further illuminated the sensitivity of the Hippo pathway to heat shock stress, which regulates YAP/TAZ activity in a LATS-dependent manner. This is achieved by promoting LATS dephosphorylation through interactions with HSP90 and protein phosphatase 5 (PP5), while concurrently fostering LATS ubiquitination and degradation to activate YAP/TAZ [92]. Osmotic stress can induce transient nuclear translocation of YAP, a process facilitated by nemo-like kinase (NLK) kinase, which phosphorylates YAP at Ser128, consequently disrupting its interaction with 14-3-3 [93].

In addition to physical and chemical cues, YAP/TAZ respond to an array of bioactive signals, mainly in the form of cytokines. Indeed, the Hippo-YAP/TAZ pathway interplay with multiple cytokine-regulated pathways, including RAS-RAF-MAPK [103,104], GPCR [73], Notch [105], canonical Wnt3a–β-catenin [82], alternative Wnt5a/b-FZD-ROR1/2 [75], TGF-β-SMAD [106], platelet-derived growth factor (PDGF)-PDGFR [107], IL6-gp130 [84], and neuregulin-1(NRG1)-ROR1-HER3 [42] pathways. For instance, the RAS-RAF-MAPK pathway operates downstream of various growth factors, including epidermal growth factor (EGF), vascular endothelial growth factor (VEGF), and fibroblast growth factors (FGF), and plays a crucial role in oncogenesis. O’Neill et al. [103] were the first to demonstrate that RAF acts as a proto-oncoprotein by counteracting apoptosis, inhibiting MST2 dimerization and phosphorylation independently of its protein kinase activity. Furthermore, a combination of mathematical modeling and experimental validation has revealed the interplay between the MST and MAPK pathways. This crosstalk is governed by competitive, switch-like transitions that regulate cell proliferation, transformation, and survival [104]. In addition, in lung and breast cancers lacking the tumor suppressor RASSF1A, TGF-β signaling is aberrantly activated, promoting the YAP-SMAD interaction and nuclear translocation, which facilitates TGF-β-mediated oncogenic transcription [106]. These interrelationships suggest the pivotal role of Hippo signaling in sustaining cellular homeostasis.

It is essential to underscore that the various inputs feeding into the Hippo pathway provide insights into diverse physiological and pathological contexts. For instance, hyperosmotic stress has particular significance within kidney physiology. Notably, renal medullary cells, experiencing elevated osmotic stress compared with cortical cells, exhibit more nuclear accumulation of YAP [108]. Similarly, mesenchymal stem cells (MSCs) display distinct responses to variations in ECM stiffness. It has been shown that activation of YAP/TAZ promotes osteoblast differentiation in the presence of rigid matrices while suppressing adipocyte formation on compliant matrices [72,109,110]. In conditions like pulmonary hypertension, characterized by elevated blood pressure in lung arteries due to abnormal collagen and elastin deposition, the role of ECM stiffening in disease is pivotal [111,112]. Fibrotic disorders, spanning hepatic, pulmonary, and cardiovascular fibrosis, exhibit intensive collagen deposition, leading to increased tissue rigidity and subsequent YAP/TAZ activation [113,114]. Upon activation, YAP/TAZ perpetuate the fibrotic process by orchestrating the expression of genes involved in the cytoskeleton and ECM, thus establishing a self-sustaining cycle of tissue remodeling [115,116]. Furthermore, growing evidence posits YAP/TAZ as molecular links between fibrosis and cancer [117]. Malignant tissues typically exhibit elevated matrix stiffness compared with their healthy counterparts [118,119], thereby stimulating cell proliferation and nurturing tumor growth via YAP/TAZ activation [120,121]. Moreover, circulating tumor cells encounter mechanical cues, including shear stress [122], wherein YAP/TAZ activation in response to shear stress assumes a pivotal role, safeguarding cancer cells in circulation and facilitating subsequent metastatic colonization [123,124,125]. While body temperature tends to remain relatively stable, it emerges as a factor in specialized hyperthermia-based cancer therapies, such as liver radiofrequency ablation and hyperthermic intraperitoneal chemotherapy [92,126,127]. Beyond mechanical and physical stimuli, bioactive signals also contribute to disease pathology. For instance, the cytokine IL1β activates YAP in macrophages, thereby contributing to inflammatory conditions such as atherosclerosis [128].

## 3. Emerging Regulators of YAP/TAZ and TEAD

While YAP phosphorylation is well known to cause its cytoplasmic retention and functional inactivation, emerging studies are shedding light on additional mechanisms of YAP/TAZ-TEAD regulation (Figure 1). First, the role of nuclear mechanical forces merits attention. Although YAP/TAZ are commonly acknowledged as mechanical sensors, the prevailing view has predominantly focused on their regulation through cytoplasmic mechanisms. Recent findings, however, point to a role for nuclear mechanical properties in modulating YAP activity. Notably, the nuclear switch/sucrose nonfermentable (SWI/SNF) complex—a multisubunit chromatin remodeling assembly involved in regulating transcription, DNA repair, and cell cycle progression—has been found to hold significance [129]. It has been found that YAP associates directly with this complex by binding to the AT-rich interactive domain-containing protein 1A (ARID1A) subunit. During periods of increased mechanical stress, cellular forces are transmitted to nuclear F-actin, thereby enhancing its interaction with the ARID1A-SWI/SNF complex. This interaction leads to the liberation and activation of YAP/TAZ [130].

Second, nuclear phase separation emerges as another determinant. Phase separation enables cells to compartmentalize biochemical reactions, resulting in membraneless organelles or biomolecular condensates that dynamically adapt to environmental changes [131]. Recent investigations demonstrate that YAP/TAZ form such liquid-like condensates in the nucleus. These condensates facilitate the concentration of YAP-specific transcription factors, TEAD [108,132]. Notably, interferon-γ (IFN-γ) has been identified as an inducer of this mechanism, with implications in anti-PD-1 resistance [133]. In addition, certain tumors exhibit aberrant YAP fusion events, such as YAP—mastermind-like domain-containing protein 1 (MAMLD1) and C11ORF95-YAP in ependymomas. These fusions contribute to tumor progression by instigating oligomeric structures and nuclear condensates that concentrate transcription factors and co-activators, including bromodomain-containing protein 4 (BRD4), mediator of RNA polymerase II transcription subunit 1 (MED1), and TEAD, while concurrently excluding transcriptional repressors like the polycomb repressive complex 2 (PRC2) complex. Consequently, this gives rise to long-range enhancer-promoter interactions that amplify oncogenic transcriptional reprogramming [134]. Interestingly, cytoplasmic components of the Hippo pathway, such as LATS, NF2, AMOT, KIBRA, and sarcolemmal membrane-associated protein (SLMAP), are also susceptible to regulation by phase separation [27,43,135,136,137], which influences both individual components and their binding partners, highlighting the impact of this physical property on Hippo signaling pathway regulation.

Third, the sequestration of YAP/TAZ-TEAD complex components within the nucleus warrants attention. Initially identified as a YAP repressor, vestigial-like family member 4 (VGLL4) competes with TEADs for YAP binding, thereby sequestering YAP and exerting tumor-suppressing functions [138]. The functional importance of this YAP-VGLL4 antagonism is supported by studies indicating that the essential roles of YAP in liver and lung development can be genetically bypassed by simultaneous VGLL4 inactivation. Notably, VGLL4 inactivation has been shown to exacerbate intrahepatic cholangiocarcinoma formation in NF2-deficient livers, while ameliorating carbon tetrachloride (CCl_4_)-induced liver damage [139]. These findings underscore the importance of VGLL4-mediated transcriptional repression. In addition to the regulation by protein–protein interactions, certain lncRNAs can disrupt the YAP-TEAD interaction through lncRNA-protein binding. For instance, our work revealed that the nuclear lncRNA metastasis associated lung adenocarcinoma transcript 1 (MALAT1) directly binds TEAD to block its interaction with YAP and target gene promoters, thereby suppressing breast cancer lung metastasis [140].

In conclusion, recent studies have facilitated the classification of YAP’s regulatory mechanisms into two modules. First, cytoplasmic regulation primarily involves YAP phosphorylation, resulting in its sequestration and degradation within the cytoplasm. Second, nuclear regulation centers on transcriptional control, influenced by factors such as nuclear mechanical sensing, phase separation, and sequestration of YAP/TAZ or TEAD. This two-tier regulation illustrates the intricate and compartmentalized control of Hippo signaling activity. Understanding these dynamics may offer both mechanistic insights and therapeutic strategies.

YAP and TAZ proteins are susceptible to degradation. It has been shown that YAP undergoes ubiquitination mediated by the SCF–β-TrCP ubiquitin complex, followed by proteasomal degradation [141,142,143]. Post-translational modifications (PTMs) play a pivotal role in governing the degradation of YAP/TAZ, and a comprehensive classification of YAP/TAZ PTMs, along with their functional implications, has been systematically reviewed [17]. However, recent studies have revealed the existence of lysosome-mediated YAP degradation. One study demonstrated that inhibitor of nuclear factor kappa-B kinase subunit epsilon (IKKε)-mediated phosphorylation targets YAP for lysosomal degradation [144], while another study identified ATP6V0d2, a subunit of the lysosomal vacuolar-type H+ adenosine triphosphatase (V-ATPase), as a crucial mediator in YAP’s lysosomal degradation [145]. These findings suggest that while proteasomal degradation may be the primary mechanism, lysosomal degradation could serve as an important alternative mechanism under specific stress conditions.

## 4. YAP/TAZ-TEAD Target Genes and Context-Dependent Regulation of Their Expression

YAP and TAZ function through collaboration with transcription factors, most notably with members of the TEAD family, to activate an extensive repertoire of target genes. These genes exert critical roles in diverse functions including development, regeneration, and homeostasis [10,146]. The target genes observed in various cell types include connective tissue growth factor (CTGF) and cysteine-rich angiogenic inducer 61 (CYR61), frequently used as indicators of Hippo pathway activity [15]. Additional target genes expressed under specific tissue or pathological contexts include amphiregulin (AREG) [147,148], Wnt5a/b [75], Tet methylcytosine dioxygenase 1 (TET1) [149], NUAK2 [63], ATF4 [150], ERα [151], VGLL3 [152], CDX2 [153], MRAS [154], and BCAR4 [155].

Interestingly, components of the Hippo pathway, including NF2, AMOTL2, and LATS2, are transcriptional targets for YAP/TAZ-TEAD, indicating a negative feedback loop [156,157,158]. Moreover, the YAP target TET1 physically interacts with TEAD’s genomic DNA, prompting localized DNA demethylation, histone acetylation, and chromatin unwinding. This culminates in the transcriptional upregulation of *TEAD1* and *TEAD4*, thus forming a positive feedback loop [149]. These feedback mechanisms ensure a fast and fine-tuned response to various stimuli. Notably, the TEAD binding sites within genes are often located at distant enhancers (rather than proximate to transcriptional start sites), where YAP/TAZ recruit multiple chromatin regulators, characterized by enriched H3K27 acetylation marks, to modulate transcriptional activity [159,160,161,162]. Due to the distances involved, which can span megabases, it is challenging to definitively assign YAP/TAZ-TEAD–bound enhancers to specific target genes [163,164].

Recent research has unveiled that the YAP/TAZ-TEAD complex also engages other transcriptional cofactors, including activator protein 1 (AP1) [161,165], hematopoietically expressed homeobox (HHEX) [97], p73 [166,167,168], SMAD [169], T-box protein 3/5 (TBX3/5) [82,83], β-catenin [82,170], and nuclear AMOT [171], thus diversifying its downstream effects [6,11]. While most studies predominantly focus on genes positively regulated by YAP/TAZ, it should be noted that approximately 50% of the target genes are subjected to negative regulation. This could be attributed to the recruitment of inhibitory factors by the YAP/TAZ-TEAD complex [172,173]. For instance, the transcriptional repressor TRPS1 acts as a potent inhibitor of YAP-dependent transactivation by binding globally to YAP-TEAD’s target sites and recruiting various corepressor complexes [172].

**Figure 2:**
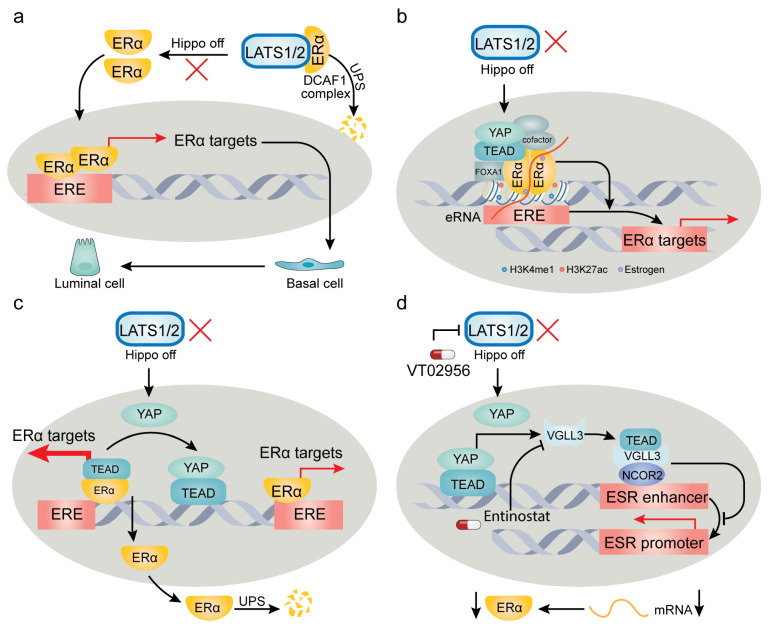
Regulation modes of YAP/TAZ on ERα. (**a**) LATS1/2 kinases interact with ERα, targeting it for ubiquitination and proteasomal degradation by the DCAF1 complex. In the absence of LATS1/2, ERα and YAP/TAZ are stabilized. (**b**) YAP-TEAD bind to a subset of ERα-bound enhancers independently of TEAD’s DNA binding ability, through a combination of factors including enhancer RNA (eRNA), the chromatin modifier FOXA1, and other cofactors, leading to H3K4 methylation and H3K27 acylation. (**c**) TEAD physically associates with ERα to enhance its occupancy of promoters/enhancers, while YAP disrupts this interaction, thereby reducing the occupancy of target promoters/enhancers by ERα and promoting ERα’s proteasomal degradation. (**d**) YAP activation stimulates the expression of VGLL3, which in turn recruits the nuclear receptor corepressor 2 (NCOR2) to the super-enhancer of the *ESR1* gene, leading to decreased ERα expression.

The intricate influence of YAP/TAZ on target genes can be exemplified by their multifaceted involvement in regulating estrogen receptor (ER) signaling. ER has been identified as a cobinding transcription factor with YAP/TEAD, playing a pivotal role in breast cancer pathogenesis. However, recent studies present divergent perspectives on how YAP/TAZ modulate ER and its functions (Figure 2). Breast cancer, originating from mammary gland epithelium, exhibits remarkable heterogeneity both in genetic profiles and histological features. The molecular subtypes—luminal A, luminal B, HER2-related, and basal-like—bear distinct prognostic implications and sensitivities to chemotherapy or targeted therapy [174]. Healthy mammary epithelium predominantly consists of luminal cells identified by markers like CK8, CK18, and E-cadherin, along with basal cells marked by CK14 and CK5/6 [175].

By using lineage tracing in genetic mouse models, Kern et al. [176] recently demonstrated that conditional codeletion of LATS1/2 kinases in mature luminal CK8+ epithelial cells fosters the development of CK14+ basal-like carcinoma. Notably, this luminal-to-basal conversion is dependent on YAP/TAZ, underscoring their role in driving breast cancer subtype transition and lineage heterogeneity [176]. Corroborating this, Furth et al. [177] revealed that LATS1 depletion in a PyMT mouse model of luminal B breast cancer promotes cancer cell plasticity, steering luminal B tumors towards a more basal-like phenotype with increased resistance to hormone therapy. On the contrary, Britschgi et al. [178] reported that LATS1/2 ablation in breast cancer cells fosters a luminal phenotype. Their findings propose that LATS1/2 kinases interact with ERα, targeting it for ubiquitination and proteasomal degradation by the DDB1- and CUL4-associated factor 1 (DCAF1) complex. In the absence of LATS1/2, they observed stabilization of ERα and YAP/TAZ, jointly controlling breast cell fate [178]. However, Ma et al. [179] contradicted this by showing the necessity of LATS1/2 in sustaining ERα expression and supporting ERα+ breast cancer growth. Their findings demonstrated that LATS1/2 deletion significantly reduces ERα levels, adversely affecting ERα+ cell proliferation without affecting ERα− cells [179]. Another study introduced a noncanonical role for YAP-TEAD, indicating their binding to a subset of ERα-bound enhancers independently of TEAD’s DNA binding capacity. This binding appears to be crucial for the activation of ERα target genes and estrogen-induced oncogenic cell growth [180].

In conclusion, these divergent findings underscore that elements of the Hippo pathway—specifically LATS1/2 and YAP/TAZ—regulate ERα through distinct mechanisms. These mechanisms can have either procancer or anticancer effects, contingent on the cancer type or subtype, cell lineage, and genetic background. The conflicting results across various studies can be attributed to several factors: (1) Kern’s study relied on genetically engineered mouse models and human organoids, while Britschgi’s study primarily used cell cultures. (2) The cell line used in Britschgi’s study, MCF10A, exhibits a basal-like phenotype while also expressing luminal markers [181], suggesting that it might not serve as an optimal model for investigating luminal-basal plasticity. (3) Ma et al. utilized MCF7 cells, characterized by ER-positivity and a luminal-like nature, unlike MCF10A cells. This underscores the impact of cell lines originating from different subtypes on both ERα and subtype marker expression. (4) Kern’s and Furth’s studies focused on the role of LATS in the luminal-basal transition by using transgenic mouse models, while the other studies focused more on the modulation of ERα activity. Although a correlation exists between ERα expression and luminal/basal subtypes, this relationship is not universally constant due to the substantial heterogeneity inherent in breast cancer [182]. These contrasting findings underscore the complexity of the influence of the Hippo pathway on breast cancer subtype transitions and its potential for both protumorigenic and antitumorigenic effects. Future research would benefit from a comprehensive approach, incorporating transgenic mice, organoids, and thoughtfully selected cell lines and xenograft models. Moreover, while the discussed studies have not explored the immunological relevance of YAP/TAZ-ERα regulation, this could present an important avenue for future investigation, particularly given the robust documentation of YAP/TAZ’s role in immune regulation in the recent literature [183] (see Section 7).

One of the pivotal questions in this field is how YAP/TAZ determine target gene specificity in response to various stimuli. Given that the Hippo pathway governs the localization and stability of YAP/TAZ, direct control over transcriptional specificity is unlikely. Recent research identifies three levels of contributing factors: (1) Tissue or cancer specificity of endogenous core YAP binding partners: particularly notable are the TEAD family, consisting of TEAD1-4, and the VGLL family, comprising VGLL1-4. While TEAD1 is ubiquitously expressed in most cancers, the expressions of TEAD2, TEAD3, and TEAD4 are more selective. These families are known to interact with varying combinations of antagonistic TEAD and VGLL family members, significantly shaping the transcriptional output [184]. Such antagonism is evident in processes like osteoblast differentiation [185] and various cancers. (2) Extrinsic inputs and context-dependent cofactors: external stimuli can activate alternative pathways, allowing other cofactors to fine-tune YAP/TAZ activity in a context-dependent manner. An example is the crosstalk between YAP/TAZ and other pathways such as TGFβ-SMAD and Wnt–β-catenin pathways. Notably, MRTF and TAZ can serve as context-dependent switches between mechanical and chemical signaling, thereby determining whether epithelial cells or fibroblasts engage in adaptive wound healing or maladaptive fibrosis responses [169]. (3) Intrinsic cofactors and tissue-specific influences: YAP/TAZ may also partner with a range of tissue- or cancer-type-specific co-factors. Transcriptional cofactors, such as AP1 [161,165], p73 [166,167], and β-catenin [82], can interact with YAP/TAZ to influence gene expression specificity. For instance, in cancers with β-catenin hyperactivation, such as colorectal cancer, β-catenin plays a crucial role in initiating the transcription of anti-apoptotic genes like *BCL2L1* and *BIRC5* through the formation of a YAP1–β-catenin–TBX5 complex [82].

## 5. Cancer-Type-Specific Alterations in the Hippo Pathway

In recent years, the pivotal role of the Hippo pathway in cancer has gained significant recognition. Aberrations in Hippo signaling have been identified across diverse cancer types, including but not limited to breast, lung, liver, prostate, and colorectal tumors [7,13,146]. Advancements in multi-omics technologies have permitted a more comprehensive and multidimensional analysis of these pathway alterations. A pancancer study, involving 9,125 patients across 33 cancer types, revealed that overall DNA aberrations in the Hippo pathway were relatively low, ranging from 1% to 5%. Particularly noteworthy is the elevated frequency of DNA amplification in *STK3* and *TAZ*, followed by *TEAD4*, *YAP1*, and *STK4* [18]. Deep deletions were predominantly observed in *LATS1/2*, underscoring its tumor-suppressing role. Concerning mutational profiles, *NF2* and *LATS2* exhibited the highest mutation rates (23.2% and 9.8%, respectively) in malignant pleural mesothelioma (MPM). Remarkably, all *NF2* mutations in MPM were truncating, resulting in diminished protein expression and functional loss. In a recent clinical trial (NCT04665206), the TEAD inhibitor VT3989 garnered attention for its antitumor efficacy in advanced mesothelioma and *NF2*-mutant cancers [186]. This serves as an exemplary instance of targeted therapy that uses mutational status to guide patient stratification. The pancancer analysis also uncovered *YAP1* gene amplification in squamous cell, cervical, lung, esophageal, head and neck, and bladder urothelial carcinomas, suggesting that these cancers might also benefit from TEAD inhibitor treatment [18,187]. Further, the researchers developed a Hippo pathway signature comprising 22 YAP/TAZ target genes [18]. This signature outperformed individual measurements, such as mRNA or protein levels of YAP/TAZ, in predicting clinical outcomes and immune microenvironment status. Subsequently, this signature has proven its efficacy as a tool for assessing Hippo pathway activity in other studies [188].

Oncogenic mutations occurring beyond the Hippo pathway have shown a correlation with the hyperactivation of YAP. A notable example lies in uveal melanoma (UM), an ocular tumor characterized by aberrant melanocyte proliferation. UM is almost universally driven by activating mutations in the heterotrimeric G-protein alpha subunits GNAQ and GNA11 [189]. These oncogenic drivers stimulate Trio-Rho/Rac signaling, which in turn promotes actin polymerization and elevates YAP activity in a Hippo-independent manner [78,190]. An unexpected discovery emerged from a recent comparative study between malignant pleural mesothelioma (MPM) and UM [191]. While YAP has a pivotal role in both cancer types, its interaction with TEAD is indispensable in MPM, but not in UM. Through systematic functional lineage analysis, this study illuminated UM’s unique rewiring of a neural-crest-derived network of melanocytic transcription factors, namely microphthalmia-associated transcription factor (MITF), SOX10, and PAX3. This network establishes a feedback loop conducive to cell proliferation [191]. These lineage-specific mechanisms explain the variation in TEAD dependence between the two cancer types, providing insights for designing targeted therapeutic approaches. For instance, TEAD inhibitors are emerging as a treatment avenue, but their applicability to UM appears to be limited.

Point mutations in *YAP1* or *TAZ* are relatively rare in most cancer types. This stands in stark contrast to more frequently mutated oncogenes like *RAS*, *PIK3CA*, and *BRAF*, as well as tumor suppressor genes such as *TP53*. Interestingly, a mechanism contributing to the hyperactivation of YAP/TAZ involves the formation of fusion proteins containing YAP/TAZ. This distinctive fusion pattern is particularly prevalent in certain uncommon cancers, including epithelioid hemangioendothelioma, ependymoma, meningioma, poroma/porocarcinoma, and nasopharyngeal carcinoma. The fusion partners in these cases are often conserved and tend to be epigenetic regulators, such as mastermind-like protein 2 (MAML2), mastermind-like domain-containing protein 1 (MAMLD1), transcription factor E3 (TFE3), and calmodulin-binding transcription activator 1 (CAMTA1) [192]. Mechanistically, most YAP/TAZ fusion proteins serve dual roles: evading regulation by upstream components of the Hippo pathway while concurrently amplifying oncogenic transcriptional activity. This amplification occurs through alterations in TEAD binding, chromatin accessibility, and the recruitment of transcription cofactors [193,194,195]. Importantly, these fusion proteins do not merely replicate the transcriptional profiles of full-length YAP/TAZ; they also introduce distinct transcriptional programs and target genes. This divergence is largely attributable to the fusion partners, which contribute additional DNA-binding properties [194,195,196]. Recent research has shed light on the role of phase separation in the function of specific fusion forms, such as YAP-MAMLD1 and C11ORF95-YAP. In ependymoma, phase separation concentrates transcription factors and coactivators, thereby enhancing oncogenic transcriptional reprogramming [134]. This study underscores the importance of altered biophysical properties induced by these aberrant fusion proteins in the dysregulation of transcriptional complexes, which could potentially pave the way for the development of targeted therapies for these specific cancer types.

## 6. Advances in YAP/TAZ-TEAD-Mediated Regulation of Tumor Dormancy, Metastatic Relapse, and Organ Tropism

**Figure 3:**
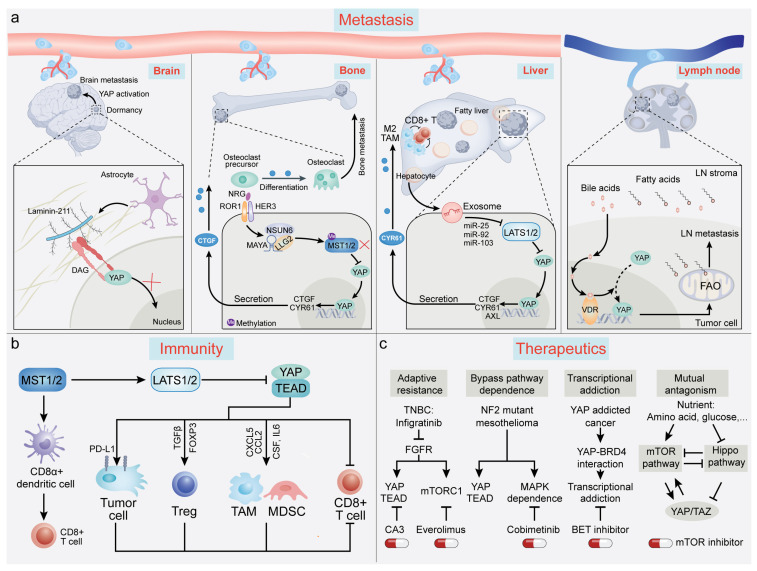
Roles of YAP/TAZ in metastasis, tumor immunity, and therapeutic response. (**a**) YAP/TAZ-TEAD-mediated regulation of tumor dormancy, metastatic relapse, and organ tropism. (1) Astrocyte-deposited laminin-211 binds the DAG1 receptor on DTCs, retaining YAP in the cytoplasm, thereby inducing cellular quiescence. (2) The lncRNA MAYA facilitates MST1 methylation and inactivation by recruiting LLGL2 and NSUN6, which leads to increased CTGF expression and secretion, promoting osteoclast differentiation and the vicious circle of bone metastasis. (3) Fatty liver conditions can boost the production of hepatocyte-derived extracellular vesicles (EVs). These EVs contain a group of microRNAs to suppress LATS, thereby enhancing YAP activity and upregulating the YAP target CYR61 in CRC cells, which nurtures an immunosuppressive milieu conducive to CRC liver metastasis. (4) Lymph nodes can selectively activate YAP in melanoma cells within their microenvironment, characterized by high fatty acid and bile acid content. This activation redirects tumor cell metabolism towards fatty acid oxidation, thereby promoting lymph node metastasis. (**b**) YAP/TAZ leads to immune suppression in cancer by promoting PD-L1 expression in tumor cells, promoting the differentiation and functions of immunosuppressive cells (e.g., Tregs, TAMs, and MDSCs), and inhibiting CD8+ T cell function. (**c**) The integration of approaches such as synthetic lethality, transcriptional addiction, and modulation of bypass pathways could produce a synergistic therapeutic impact.

Over 90% of cancer-related fatalities result from metastasis. Unfortunately, viable treatments for patients with metastatic disease remain scarce [21,197,198]. Since the initial identification of YAP as a prometastatic factor [34,199], YAP and TAZ have been recognized as drivers at various stages of the metastatic process, as discussed previously [22,200,201]. In this review, we focus on providing up-to-date insights into the involvement of YAP/TAZ in tumor dormancy, metastatic relapse, and organ tropism (Figure 3a).

Dormant tumor cells serve as the precursor for future recurrences. These cells differ from actively dividing cells in several aspects, including cell cycle quiescence, resistance to anoikis (cell death due to detachment from the ECM), and enhanced stem cell-like properties [202,203]. Various events cause tumor cells to enter a dormant state, with emerging research indicating the pivotal involvement of YAP/TAZ in maintaining this dormancy and governing subsequent relapse. Specifically, low YAP/TAZ activity is necessary to keep disseminated tumor cells (DTCs) in a quiescent or dormant state. This aligns with the notion that during dormancy, cells encounter considerable stress and necessitate restrained proliferation for survival. A current focus in this area is comprehending the mechanisms guiding cells to exit from this dormant state once they encounter a more favorable milieu [202,203].

The dynamic interplay between ECM components and their cell-surface receptors governs tumor dormancy, exerting either a sustaining or disruptive influence on the quiescent state in a context-dependent manner [204,205,206]. Recent findings underscore the involvement of YAP/TAZ in orchestrating these interactions. For example, astrocyte-deposited laminin-211 has demonstrated the ability to bind the dystroglycan receptor (encoded by *DAG1*) on breast cancer DTCs. This binding retains YAP in the cytoplasm, thereby inducing cellular quiescence. Disruption of the laminin-211–DAG binding allows YAP to translocate into the nucleus, resulting in TEAD-dependent transcription and metastatic colonization of the brain [207]. Similarly, Er et al. [208] demonstrated that DTCs employ cell adhesion molecule L1 (L1CAM) to facilitate pericyte-like spreading on capillaries. This process triggers the activation of YAP and myocardin-related transcription factor (MRTF) by engaging β1 integrin and integrin-linked kinase (ILK), thereby enabling metastatic outgrowth in target organs [208]. Collectively, these studies underscore the critical role of YAP in steering DTCs through the transition from a dormant to a proliferative state upon arrival at a favorable niche.

The concept of tumor dormancy extends beyond the scope of DTCs and is markedly influenced by various stressors, such as transient mitotic arrest induced by chemotherapy. A recent study by Ohta et al. [209] revealed that in colorectal cancer, LGR5+ cancer stem cells exhibit dormancy marked by p27 positivity in a chemo-naïve state. In these cells, the upregulation of collagen alpha-1(XVII) chain (COL17A1), a cell-adhesion molecule, is crucial for maintaining cancer cell dormancy. Chemotherapy disrupts this quiescent state by initiating proteolysis of COL17A1, thereby activating FAK-YAP signaling [209]. This work underscores the essential role of YAP inactivation in sustaining dormancy, a finding consistent with earlier studies on dormant DTCs [207,208]. On the other hand, however, a study by Kurppa et al. [210] suggests that YAP activity is essential for conferring resistance to combined EGFR-MEK inhibition, thereby inducing dormancy in an EGFR-mutated non-small cell lung cancer model. Mechanistically, YAP-TEAD activation downregulates the expression of apoptosis-related genes (such as *BMF* and *BIM*), consequently amplifying prosurvival signals [210]. These ostensibly conflicting findings could potentially be attributed to varying tissue origins, therapeutic strategies, and YAP expression levels. In Kurppa’s study, the complete knockout of YAP through Clustered Regularly Interspaced Short Palindromic Repeats (CRISPR) technology might have yielded phenotypes influenced not only by the inhibition of drug-induced dormancy, but also by alterations in genes vital for basic cellular functions. In Ohta’s study, a reduction in YAP levels in dormant cells does not necessarily negate YAP’s relevance in maintaining dormancy, since a baseline level of YAP activity might be necessary to suppress apoptosis. Thus, it becomes evident that YAP’s contribution to tumor dormancy is context-dependent. Cancer cells may adapt to challenging environments by fine-tuning YAP expression or activity to favor prosurvival and anti-apoptotic states.

Organotropism, the propensity of cancer cells to preferentially metastasize to specific organs or tissues, is governed by intrinsic cellular characteristics and extrinsic cues from the microenvironment [21,211]. YAP/TAZ, central conduits for environmental sensing and integration (as detailed in Section 2), appear to contribute to organotropism determination. Recent evidence suggests that YAP/TAZ exert an influence on metastasis in various tissues, including the liver [212,213,214], brain [207], bone [42], lymph node [101], and peritoneum [215], albeit in a context-dependent manner. For instance, sentinel lymph nodes, critical hubs for subsequent tumor dissemination, can selectively activate YAP in melanoma cells within their microenvironment, characterized by high fatty acid and bile acid content. This activation redirects tumor cell metabolism towards fatty acid oxidation, thereby promoting lymph node metastasis [101]. In colorectal cancer (CRC) treatment, liver metastasis poses a formidable obstacle. Recent research by Wang et al. [212] uncovered that fatty liver conditions can boost the production of hepatocyte-derived extracellular vesicles (EVs). These EVs subsequently enhance YAP activity and upregulate the YAP target CYR61 in CRC cells, thus nurturing an immunosuppressive milieu conducive to CRC liver metastasis [212]. In breast cancer, a recently identified lncRNA known as MAYA can act as an adaptor, recruiting LLGL and NSUN6 to facilitate MST1 inactivation by directly mediating the methylation of MST1 at Lys59. The inactivation of MST1 results in increased expression and secretion of the YAP target CTGF, thus promoting bone metastasis [42] (Figure 3a). Furthermore, high-throughput sequencing has illuminated the link between YAP/TAZ activation and accelerated brain metastasis in patients with lung adenocarcinoma [216]. This correlation is corroborated by another study demonstrating increased *YAP1* amplification frequencies in a cohort of lung adenocarcinoma patients with brain metastasis [217]. While these bioinformatic analyses require further experimental validation and mechanistic dissection, the growing body of research emphasizes the pivotal role of YAP/TAZ in dictating organ-specific metastasis, accomplished through interactions of tumor cells with both premetastatic and metastatic niches.

## 7. Advances in YAP/TAZ-TEAD-Mediated Regulation of Tumor Immunity

Since its discovery in the 1990s, early research on the Hippo-YAP pathway predominantly centered on its cell-autonomous functions that are independent of immune regulation. It was not until the past decade that the significance of YAP/TAZ in tumor immunity gained recognition, marked by a surge of findings in the last five years [9] (Figure 3b). Tumor cells employ diverse mechanisms to evade immune surveillance, including suppression of antigen presentation, upregulation of immune checkpoint ligands such as PD-L1, and secretion of specific cytokines to recruit immunosuppressive cell populations. Collectively, these strategies create a tumor microenvironment (TME) that is inhospitable to the recruitment and functionality of effector T cells [21,218].

YAP/TAZ have diverse roles in the regulation of tumor immunity. Although initial investigations suggested that depletion of LATS1/2 (thus activating YAP/TAZ) enhances cancer immunity [219], a growing body of evidence now supports that, on the whole, YAP/TAZ function as suppressors of antitumor immunity [220]. YAP/TAZ can transcriptionally activate PD-L1, thereby inhibiting T cell-mediated cytotoxicity [221,222,223,224]. Moreover, YAP/TAZ mobilize myeloid-derived suppressor cells (MDSCs) and tumor-associated macrophages (TAMs) by upregulating various cytokines, including C-X-C motif chemokine 5 (CXCL5), C-C motif chemokine 2 (CCL2), macrophage colony-stimulating factor 1 (CSF1), and interleukin-6 (IL6) [225,226,227]. Interestingly, YAP is upregulated in regulatory T cells (Tregs), elevating the expression of the pivotal Treg transcription factor, forkhead box protein P3 (FOXP3). This is governed by YAP-dependent enhancement of activin signaling, consequently amplifying TGFβ-SMAD signaling in Tregs. Genetic inactivation of YAP substantially diminishes the immunosuppressive potential of Tregs in vivo and improves the efficacy of anti-PD-1 treatment [228]. Notably, YAP can also act as an immunosuppressive factor that inhibits effector T cell differentiation, and in vivo experiments have demonstrated that ablation of YAP augments T-cell responses [229]. Further, YAP activation is implicated in resistance to anti-PD-1 immunotherapy. Mechanistically, following anti-PD-1 treatment, IFN-γ induces phase separation of YAP in tumor cells, thereby establishing a transcriptional hub that recruits multiple epigenetic modifiers to amplify the expression of targets (such as CD155), ultimately promoting anti-PD-1 resistance [133]. Beyond its effects on T cells, recent research has shown that dendritic cell activity is governed in a manner that is dependent on MST1/2 but is independent of LATS1/2 and YAP/TAZ. Deletion of MST1/2 selectively impairs the ability of BAF3+CD8α+ dendritic cells to activate CD8+ T cells due to disruption of mitochondrial metabolism dynamics and crosstalk to non-canonical nuclear factor-κB (NF-κB) signaling [230].

In addition to their role in modulating adaptive immunity, YAP/TAZ exert influence on innate immune responses. Typically, host cells detect viral RNA and DNA through various pattern-recognition receptors. These receptors then recruit adaptor proteins to activate TANK-binding kinase 1 (TBK1) and/or IKKε kinases, which in turn phosphorylate interferon regulatory factor 3 (IRF3) and NF-κB. This results in their nuclear translocation and the subsequent transcriptional activation of inflammatory cytokines, including type I interferons (IFN-α and IFN-β) [231]. YAP can inhibit IRF3 dimerization and nuclear translocation, thereby dampening the antiviral IFN-β response [144]. Moreover, YAP/TAZ can directly bind to TBK1 and IKKε kinases, thus inhibiting their Lys63-linked ubiquitination and subsequent activation in response to cytosolic nucleic acid sensing [232]. In addition, YAP-mediated suppression of the TBK1-IRF3 pathway is positively regulated by serine metabolism [145].

IRF3 is a key effector in the cGAS-STING pathway, and recent work has revealed that a specific mutation in the FERM domain of NF2 (NF2m) transforms NF2 into a potent suppressor of the cGAS-STING pathway. This is mediated by the formation of NF2m-IRF3 condensates through phase separation [233]. In mutant NF2 cells, on the one hand, YAP is activated and impairs IRF3-mediated innate immunity; on the other hand, NF2m-IRF3 condensate formation also inhibits the cGAS-STING pathway [233]. Interestingly, the regulation of IRF3 by YAP/TAZ extends to cancer immunity. Contradicting the aforementioned studies [144,232], Jiao et al. [234] demonstrated that viral infections trigger IRF3-mediated nuclear translocation of YAP. In this particular context, IRF3 forms complexes with YAP and TEAD, augmenting their transcriptional activity. This study further unveiled that IRF3 acts as an enhancer of the YAP-TEAD axis in gastric cancer. Pharmacological inhibition of IRF3 using amlexanox was found to inhibit YAP-driven gastric tumor growth [234].

## 8. Advances in YAP/TAZ-TEAD-Mediated Regulation of Therapeutic Response

Despite advancements in cancer therapies, resistance remains a substantial challenge. Numerous studies have indicated that YAP/TAZ play pivotal roles in mediating both intrinsic and acquired resistance to chemotherapy, targeted therapy, and immunotherapy. This has been comprehensively summarized in several excellent reviews [14,235,236,237,238,239,240]. In this context, we highlight several studies that explored strategies for conquering therapy resistance by leveraging mechanisms of adaptive resistance, oncogenic dependence, transcriptional addiction, and pathway crosstalk (Figure 3c).

A recent study reported fibroblast growth factor receptor (*FGFR*) amplification in 10–15% of triple-negative breast cancer (TNBC) cases [241]. Although various FGFR inhibitors have undergone clinical trials for TNBC, adaptive resistance frequently undermines their therapeutic efficacy. Intriguingly, the YAP-TEAD complex plays a vital role in this resistance. Through BRG1-dependent chromatin remodeling, YAP-TEAD activates YAP-associated enhancers. This activation fosters amino acid transport, a signal sensed by the mTORC1 pathway. Aligning with this mechanism, the combined use of mTORC1 or YAP-TEAD inhibitor with FGFR inhibitor has been shown to synergistically suppress TNBC growth in patient-derived xenograft models [241].

By using computational pancancer machine-learning methodologies to analyze transcriptional profiles, Pham et al. [187] found a significant correlation between YAP/TAZ dependence and MAPK pathway activity. Across diverse tissue lineages and histological cell types, mesothelioma cells emerged as particularly reliant on YAP/TAZ. In contrast, hematological cell lines exhibited low dependence on YAP/TAZ activity [187]. Further investigation through chemical screening of small-molecule compounds revealed combining MEK inhibitors with YAP1 knockdown significantly improved responsiveness in *YAP1*-amplified cancer cell lines [187]. This finding underscores the pivotal role of crosstalk between alternative signaling pathways in designing therapeutic strategies for treating *YAP1*-dependent cancers. Beyond *YAP1*-amplified malignancies, the concurrent inhibition of YAP-TEAD and MEK-RAF pathways demonstrated synthetic lethality in tumors harboring *BRAF* or *RAS* mutations, suggesting the promise of this strategy across a wide range of cancers, particularly melanoma and non-small cell lung cancer [154,242].

Another insight gained from recent research underscores the role of YAP/TAZ in orchestrating transcriptional addiction in specific cancer types. The term “oncogene addiction” denotes the phenomenon wherein cancer cells become overwhelmingly dependent on specific oncogenes for their continuous proliferation and progression [243]. This concept has evolved to incorporate aberrant transcriptional programs, including transcriptional dysregulation, enhancer malfunction, and epigenetic modulation, thereby giving rise to the notion of “transcriptional addiction” [244]. A study by Zanconato et al. [162] demonstrated that YAP/TAZ contribute to transcriptional addiction by directly interacting with the transcriptional coactivator bromodomain-containing protein 4 (BRD4). This interaction facilitates the recruitment of both BRD4 and RNA polymerase II, thereby amplifying the expression of an array of growth-stimulating genes located at YAP/TAZ-associated enhancers and promoters. Importantly, treatment with BET inhibitors, which target BRD2, BRD3, and BRD4, effectively reverses the protumorigenic activity and drug resistance driven by YAP/TAZ [162].

The mammalian target of rapamycin complex 1 (mTORC1) signaling pathway, essential for sensing metabolic environment, has recently been reported to interconnect with the Hippo pathway, ensuring precise regulation of cell behavior [245]. YAP/TAZ have a critical role in amino acid-induced mTORC1 activation, particularly under nutrient-restrictive conditions [246]. Conversely, the mTORC1 pathway diminishes the Hippo pathway activity, thereby enhancing YAP/TAZ-driven gene expression [247]. Through genome-wide CRISPR screening, Dai et al. [248] interrogated vulnerabilities in TNBC and identified an interplay between mTOR and Hippo pathways. Notably, pharmacological inhibition of mTOR and YAP/TAZ reduced TNBC growth. This study suggests that the reciprocal regulation between mTOR and YAP/TAZ pathways holds translational potential in TNBC, a breast-cancer subtype that lacks effective targeted therapies.

Collectively, these studies indicate that harnessing the attributes of YAP/TAZ dependence, synthetic lethality, transcriptional addiction, and YAP/TAZ-mediated pathway crosstalk and activation could serve as innovative avenues for developing synergistic therapeutic approaches.

## 9. Emerging Roles of YAP/TAZ in Tumor Suppression

**Figure 4:**
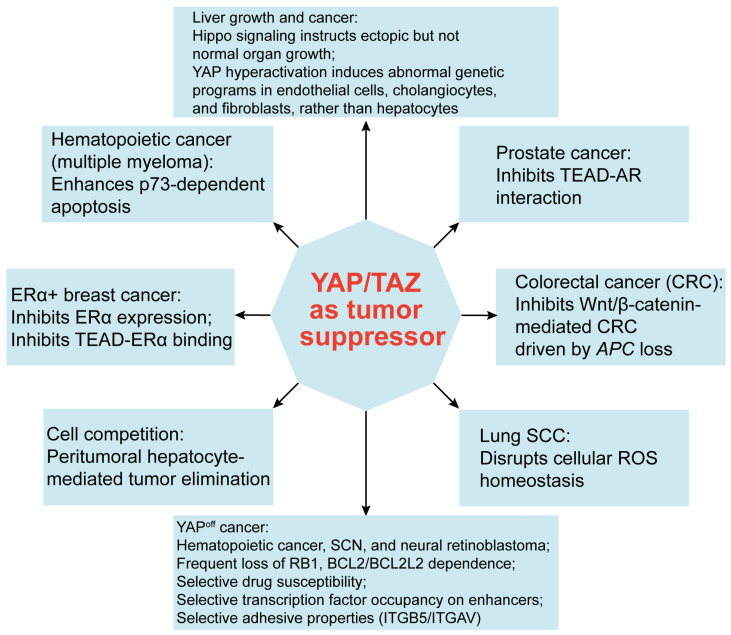
The tumor-suppressing role of YAP/TAZ. Summary of recent research on the tumor-suppressing role of YAP/TAZ and the underlying mechanisms.

In contrast to conventional understanding, the activation of YAP/TAZ has been shown to elicit tumor-suppressing effects in specific contexts [249] (Figure 4). This observation may appear counterintuitive, given the well-established role of YAP/TAZ in promoting the survival and proliferation of tumor cells. However, early research sporadically documented this anticancer role. For instance, p73, an early-identified cofactor of YAP, has demonstrated the ability to induce apoptosis in multiple myeloma, a form of hematopoietic cancer [156,157,232]. Later, it was found that in mammary tumor cells, elevated YAP activity inhibits the expression of ERα, a driver of ERα-positive breast cancer growth [151,152,179].

Mechanistically, while TEAD physically associates with ERα to enhance its occupancy of promoters/enhancers, YAP disrupts this interaction, thereby reducing the occupancy of target promoters/enhancers by ERα and promoting ERα’s proteasomal degradation [151]. Concurrently, YAP activation stimulates the expression of VGLL3, which in turn recruits the nuclear receptor corepressor 2 (NCOR2) to the super-enhancer of the *ESR1* gene [152]. Interestingly, a similar mechanism appears to be shared by another hormone receptor, androgen receptor (AR), as elevated nuclear YAP has been found to disrupt the TEAD-AR interaction and inhibit AR’s transcriptional activity, thereby acting as a context-dependent tumor suppressor in AR+ prostate cancer [250]. Beyond breast cancer and prostate cancer, YAP/TAZ activation following LATS1/2 deletion in hepatocytes triggers widespread p53-dependent cellular senescence and death [251]. In addition, YAP activation has been found to disrupt the homeostasis of reactive oxygen species (ROS), thereby impeding tumor growth in lung squamous cell carcinoma (SCC) via a ROS-mediated mechanism [252,253].

In the context of CRC, Cheung et al. [254] used multiple mouse models—including MST1/2 deletion, LATS1/2 deletion, and YAP activation—to demonstrate that YAP activation inhibits tumor growth in APC loss-induced CRCs. YAP/TAZ appear to constrain Wnt signaling through multiple mechanisms, consequently shifting LGR5+ CRC stem cells towards a less aggressive, regenerative cell state conducive to wound healing [75,254,255,256,257]. These findings illuminate YAP/TAZ’s counteractive role against Wnt signaling, a critical pathway in CRC. Overall, the tumor-suppressing effects of YAP/TAZ seem to mainly manifest in hematological cancers, ERα-positive breast cancer, Wnt/β-catenin-driven CRCs, and lung SCCs. These effects are mediated by diverse context-specific mechanisms.

A recent study by Pearson et al. [23] combined bioinformatic analysis and genetic model validation to establish a binary pan-cancer classification. This investigation uncovered the segregation of 1036 cell lines in the Cancer Cell Line Encyclopedia (CCLE) database into two groups, termed YAP^on^ and YAP^off^, through principal component analysis of the transcriptome [23]. This categorization was subsequently extended to primary cancers in the TCGA/TARGET database, defining YAP^on^ and YAP^off^ pancancer states. Notably, YAP^off^ cancers are enriched in hematopoietic cancers (including multiple myeloma), neural retinoblastoma, and small-cell neuroendocrine cancers. A recent study experimentally validated that in small-cell lung cancer (SCLC), YAP loss is required for the maintenance of the neuroendocrine (NE) feature. Intriguingly, ectopic overexpression of YAP induced NE to non-NE conversion, leading to acquired chemoresistance [258]. In addition, YAP^off^ cancers frequently exhibit loss of the tumor suppressor RB1. In contrast, YAP^on^ cancers are enriched in other solid tumor types and are characterized by the prevalence of wild-type RB1 [23].

By dividing cell lines in two public drug databases (the CTRP and GDSC databases) into YAP^on^ and YAP^off^ groups, Pearson et al. [23] conducted an indepth analysis of high-throughput drug sensitivity. The common hits in both databases showed that YAP^off^ cancers displayed high susceptibility to inhibitors targeting NAMPT, a NAD synthesis enzyme, as well as aurora kinases, the anti-apoptotic BCL family, and histone deacetylases, thereby revealing a broader YAP^off^ selectivity. On the other hand, YAP^on^ cancers exhibited increased sensitivity to inhibitors targeting tyrosine kinases (e.g., SRC, ERBB, and EGFR) or serine/threonine kinases (e.g., BRAF).

Leveraging the Cancer Dependency Map (DepMap) database, Pearson et al. [23] used functional genomics to define essential genes across hundreds of cancer cell lines. It was observed that YAP^off^ cancers demonstrated significant dependence on anti-apoptotic *BCL2* and/or *BCL2L2* genes. Moreover, the overall dependence gene sets were enriched in metabolic enzymes, including purine synthesis, displaying a substantial overlap with MYC/MAX-bound targets. On the other hand, YAP^on^ gene dependencies were enriched in regulators of integrin, ECM, and cytoskeletal pathways. Consistent with this ECM signature, which correlates with cell adherence, YAP^off^ cancer cell lines exhibited a propensity for nonadherent or semi-adherent behavior, whereas YAP^on^ cell lines displayed predominantly adherent characteristics. The authors validated that YAP suppresses YAP^off^ cancers through TEAD binding and gene activation, mirroring the oncogenic mechanism observed in YAP^on^ cancers. Further, based on genomic analysis of enhancers to which TEAD recruits YAP for the induction of cell-cycle genes, YAP^on^ enhancers mainly harbored motifs for AP1 (JUN/FOS/ATF family), MafA, FOXM1, or REST. In contrast, YAP^off^ enhancers lacked AP1 binding sites but exhibited enrichment in motifs for bHLH (ASCL1/TCF12/MAX) and homeobox (OTX2/PBX3/LHX2/NKX2-1) transcription factors. This distinctive regulation of enhancers potentially governs downstream gene expression, contributing to divergent roles of YAP in cell growth.

Moreover, via CRISPR screening, specific genes mediating YAP-induced cell growth inhibition were identified. Among these, integrin beta-5 (ITGB5) and its partner integrin alpha-V (ITGAV) were recognized, as they mediate cell–matrix adhesion and play a procancer role in YAP^on^ cancers, while exhibiting an opposing effect by suppressing cell growth in YAP^off^ cancers. In conclusion, YAP^off^ and YAP^on^ cancers exhibit distinct drug vulnerabilities, genetic dependencies, distinct enhancer dynamics, and opposing adhesive properties, allowing for the functional stratification of binary cancer categories. These distinctions offer insights for tailored therapeutic interventions [23].

Beyond the intrinsic tumor-suppressing functions of YAP/TAZ, recent studies have unveiled their non-cell-autonomous roles in cancer suppression, particularly at the interface between tumor cells and adjacent normal tissues. Moya et al. [259] used a genetically engineered mouse model of liver cancer to investigate this phenomenon, finding that adjacent normal hepatocytes have a tumor-suppressing function, actively restraining tumor growth. Intriguingly, experimentally induced hyperactivation of YAP in these peritumoral hepatocytes not only triggered the regression of primary liver tumors but also exerted the same effect on liver metastases originating from melanoma [259]. This compelling line of research underscores the significance of the relative activity of YAP/TAZ between tumor cells and their adjacent tissues. It also draws attention to the concept of cell competition-mediated tumor elimination, a phenomenon initially observed in *Drosophila* [260,261]. The relevance of this concept has grown evident, exerting a substantial influence on tumorigenesis and tumor progression, as supported by various studies [262,263,264].

By using genetic models of mouse liver growth and drosophila imaginal discs, which serve as developmental paradigms for probing the Hippo pathway and organ growth, Kowalczyk et al. [188] found that Hippo signaling does not instruct normal growth, and the overgrowth phenotypes induced by this pathway are caused by the activation of abnormal genetic programs. Specifically, YAP/TAZ are not required for hepatoblast proliferation but required for their differentiation into cholangiocytes. Remarkably, excessive YAP activation in adult hepatocytes does not cause liver overgrowth because it activates a normal progenitor (hepatoblast) cell program. Rather, YAP hyperactivation ectopically induces abnormal genetic programs of endothelial cells, cholangiocytes, and fibroblasts, as well as other genes that are not normally expressed in hepatocytes. A similar Yap/Taz working pattern was observed in the development of drosophila eye discs. These findings rectify a long-standing misconception about the role of Hippo signaling in organ growth and prompt a re-evaluation of the understanding of these functions in cancer and regeneration.

In summary, recent discoveries regarding YAP/TAZ’s tumor-suppressing roles offer a nuanced, context-dependent perspective for comprehending their authentic roles in tumor progression. Experimental design considerations should include potential pitfalls. For instance, the activation of YAP/TAZ through LATS1/2 knockout models could inadvertently result in supraphysiological YAP/TAZ activity, triggering unforeseeable outcomes such as disruption of the TEAD-ER interaction. Moreover, the contribution of endogenous LATS cofactors, such as p53 regulation, warrants more attention. Regarding therapeutic implications, promising avenues beckon exploration. In the context of YAP^off^ cancers, strategies aiming to enhance YAP/TAZ activity hold notable potential. This objective could be better achieved by targeting upstream MST and LATS kinases, which are more amenable to pharmacological intervention. For YAP^on^ tumors, targeted inhibition of YAP-TEAD activity in tumor tissues should be carefully calibrated. The goal here is to administer a dosage potent enough to eliminate cancer cells while preserving the competitive advantage conferred by adjacent normal tissues.

## 10. Conclusions and Future Directions

Recognizing the pivotal role of YAP/TAZ as oncogenic drivers, researchers have made substantial progress in elucidating their downstream tumorigenic processes and the upstream regulatory mechanisms. This burgeoning knowledge has prompted dedicated efforts to develop therapeutic strategies targeting this pathway, as comprehensively reviewed in the recent literature [7,8,9,192,201,265,266]. In general, YAP/TAZ are considered challenging to target with drugs, and therapeutic approaches primarily focus on their cofactor TEAD, employing two main strategies [8]. (1) Targeting TEAD palmitoylation in its central pocket: TEAD undergoes autopalmitoylation at a conserved cysteine residue, which is essential for its interaction with YAP/TAZ and its transcriptional activity. Covalently tethered palmitate on TEAD is nestled within a deep hydrophobic pocket. The combination of computer-based modeling and chemical library screening has led to the identification of TEAD inhibitors that block this palmitoylation, including VT3989, VT107, flufenamic acid, vinylsulfonamide, and MGH-CP1. VT3989 has progressed to clinical trials and has shown promising antitumor effects in patients with malignant mesothelioma and other advanced tumors harboring *NF2* mutations [186]. (2) Disrupting the TEAD-YAP/TAZ binding interface: The YAP-TEAD complex involves three binding interfaces, and small-molecule inhibitors have been developed to disrupt these interfaces. Notable inhibitors in this category include aurintricarboxylic acid, Super-TFU, and peptide 17 [8].

With promising clinical trials currently in progress, several key considerations should be addressed for future clinical translation: (1) Patient stratification: Presently, YAP/TAZ-targeting therapy finds utmost relevance in tumors carrying *NF2* mutations, such as mesothelioma, primarily because these mutations lead to YAP/TAZ hyperactivation. However, the size of this patient population is relatively small. Hence, the quest for more refined markers or criteria is vital for including a broader target population. (2) Combination therapies: For certain types of cancer, combination therapy should be considered. The strategic integration of approaches such as synthetic lethality, transcriptional addiction, and modulation of bypass pathways could produce a synergistic therapeutic impact. (3) Immune regulation: While the understanding of the role of the Hippo pathway in immune regulation is unfolding, initial basic research outcomes are encouraging. Advancing translational inquiries is essential to enriching our insights and pinpointing potential therapeutic targets. (4) Tumor suppressive roles: Recent studies have unveiled the tumor-suppressing facet of YAP/TAZ in certain contexts. In these instances, targeting upstream kinases, notably, LATS, presents a rational avenue, given that kinases are generally amenable to pharmacological targeting. Several LATS inhibitors, such as VT02956, TRULI, and GA-017, are available, and further investigation into their role in targeting specific cancer types where YAP/TAZ exhibit a tumor-suppressive role is underway. (5) Intratumoral heterogeneity: Rigorous scrutiny of intratumoral heterogeneity stands as a prerequisite due to its profound impact on therapeutic outcomes and its contribution to the emergence of treatment resistance. (6) Drug resistance: While drugs aimed at disrupting the YAP/TAZ–TEAD interaction are in developmental stages, preliminary investigations have indicated the potential for acquired resistance [267]. Strategies for overcoming or mitigating such drug resistance will be of paramount importance in future research endeavors.
